# ‘We need a warm hug to remind us that we are loved’: a qualitative study of psychosocial health and wellbeing among lesbian forced migrants

**DOI:** 10.1186/s12889-026-26240-8

**Published:** 2026-01-17

**Authors:** Maria Gottvall, Rummage Isaac, Ronah Ainembabazi, Osszián Péter-Szabó, Tommy Carlsson

**Affiliations:** 1 The Department of Health Sciences, The Swedish Red Cross University, Huddinge, Sweden; 2 CIRCLE – Complex Intervention Research in Health and Care, The Department of Women’s and Children’s Health, Uppsala university, Uppsala, SE-751 85 Sweden

**Keywords:** Asylum seekers, Lesbian, Forced migrants, Mental health, Sexual and gender minorities

## Abstract

**Background:**

Seeking refuge in another country is a significant life event associated with health burdens and unmet health needs. However, the diversity within the migrant population necessitates a multidimensional framework acknowledging intersectional perspectives, and there is a notable lack of studies about the health and support of sexual minority forced migrant women. The aim of this study was to explore psychosocial health and wellbeing among lesbian forced migrants in Sweden.

**Methods:**

Exploratory qualitative study consisting of semi-structured interviews with lesbian forced migrants recruited via purposeful, convenience, and snowball sampling. All participants originated from Africa. Data were analyzed with systematic text condensation through a collaborative approach involving researchers, experts by lived experience, and a clinical psychologist.

**Results:**

Participants experienced a challenging journey involving intersectional disadvantages and an unsafe living situation. They struggled to meet basic needs, experienced painful memories during asylum interviews, feared potential deportation, and faced loneliness in a new and unfamiliar society. A newfound freedom and social support empowered them to keep pursuing happiness in the face of struggles. They found strength in welcoming spaces, found belonging and comfort among peers, and were aided through informational and instrumental support. When trying to access health services, participants suffered in silence because of barriers hindering their access. While the importance of sensitivity and respect in clinical settings was emphasized, non-affirming behaviors were sometimes encountered when interacting with health professionals. Interpreters’ discretion and safety were considered essential aspects when they are utilized in health services.

**Conclusions:**

Lesbian forced migrants face a range of challenges impacting their health and wellbeing. Loneliness is a pressing concern, while social support among peers is highly desired and appreciated. Lesbian forced migrants emphasize the importance of accessible and affirming health services, including respectful behaviors and appropriate utilization of interpreters promoting client safety. Peer support has the potential to offer comfort and belonging, but more research is needed.

**Supplementary Information:**

The online version contains supplementary material available at 10.1186/s12889-026-26240-8.

## Background

In many countries, sexual minority individuals face considerable oppression [1] and lack protection of basic human rights [2]. Violence against sexual minority individuals is enacted by a range of perpetrators and takes many severe forms, including torture, conversion attempts, sexual violence, and physical assault [1, 3]. Structural stigmatization and victimization of sexual minority individuals can induce minority stress [4]. The concept is widely recognized as a model of the health disparities observed between sexual minority individuals and heterosexual counterparts [5–7]. Repeatedly, studies show high prevalence of depression, anxiety, suicidality, and substance abuse among sexual minority individuals [5, 7]. However, there is a lack of research about the health of lesbian women and how to tailor health services accordingly [5].

Severe oppression and persecution against sexual minority individuals can result in no other option than to flee and seek asylum in another country [1]. Being forced to migrate is a significant life event associated with the development of health burdens. Mental disorders and physical diseases are disproportionately more prevalent among forced migrants [8, 9]. However, the diversity within the migrant population necessitates a multidimensional framework acknowledging intersectional perspectives [10]. Research underscores the influence that gender has on health during migration [11, 12] and resettlement [13, 14], indicating that women and gender diverse populations experience unique health burdens and challenges. Taken together, the observed structural health inequities highlight a need to address gender disparities observed within the population of forced migrants [15, 16].

Self-identifying as a woman and a lesbian can introduce unique stressors impacting the health of forced migrants [17]. While countries granting asylum to lesbians facing persecution save their lives, the asylum process itself can be stressful and uncertain [18, 19]. Furthermore, sexual minority forced migrants face a range of stressors when trying to transition into the host country society [1, 17, 20]. Intersectionality concerns how power and privilege are shaped by the overlap of identities and positions in society, acknowledging the multidimensional and complex circumstances influencing the health and wellbeing of individuals [21]. Through an intersectional perspective, being a lesbian woman may lead to unique disadvantages and invisibility related to power structures favoring cisgender men. Limited research calls attention to the dual discrimination and disbelief experienced by lesbians seeking asylum [22], illustrating challenges in talking openly about sensitive topics and needing to conform to stereotypes [23].

While the field of research about sexual minority forced migrants is expanding, there is a notable lack of qualitative studies focusing on the health and support of women and gender diverse persons self-identifying as lesbian [3, 17]. Oftentimes, studies present results in which lesbian forced migrants are grouped together with other more represented subpopulations, such as gay men [17]. Limited empirical research call attention to discrimination and social exclusion based on several grounds, as community spaces are focused on the needs of more privileged subgroups [24]. Furthermore, there is a dearth of research addressing the health and wellbeing of lesbian forced migrants beyond the legal aspects of seeking asylum. Thus, this study set out to bridge that gap by exploring psychosocial health and wellbeing among lesbian forced migrants. Specifically, the study addressed post-migration experiences related to three areas: (i) psychosocial challenges, (ii) social support, and (iii) support from health services. Herein, we applied an inclusive definition of the concept lesbian [25], encompassing women and gender diverse individuals who view themselves as lesbian with a physical, romantic, and/or spiritual attraction to women.

## Methods

### Study setting

This study was conducted in Sweden, which acknowledges persecution based on sexual and gender minority status as a valid reason for asylum. Sweden has no criminalization of same-sex consensual sexual acts between adults and has implemented various laws protecting the rights of sexual and gender minority individuals [2]. At the time of this study, adult asylum seekers and undocumented migrants had the right to access healthcare that could not be deferred. Moreover, asylum seekers could choose to be placed in accommodations with other asylum seekers or to arrange housing through other routes at the time of this study. Various non-governmental organizations acting on local and national levels support sexual and gender minority forced migrants.

### Study design

This was an exploratory qualitative study with an inductive approach and co-analysis using public contribution in research with sexual minority forced migrants who have lived experience; henceforth referred to as experts by lived experience [26]. Public contributors acted as experts by lived experience and were engaged throughout all phases of the research cycle, ranging from conceptualization to analysis and reporting, In this study, the goal of the public contribution efforts together with the experts by lived experience was to ground the data, analysis, and reporting in the perspectives of people who represent sexual and gender minority forced migrants. Exploratory qualitative studies are suitable when in-depth understandings are needed about complex lived experiences among individuals of underrepresented groups, through open-ended approaches such as interviews [27]. All participants received a gift card of SEK 500 after the conclusion of the interview.

The theoretical underpinnings relate to intersectionality [21] and minority stress [4]. From an intersectional perspective, societal disadvantages based on multiple grounds, such as being a forced migrant and a lesbian woman simultaneously, involve a heightened risk of psychological distress [28]. The minority stress model outlines the mechanisms leading to health-related burdens among sexual and gender minority individuals, stemming from societal structures imposing stigma and marginalization [6]. While the theories aided in the rationale, an inductive approach was utilized throughout the data collection and analysis, to generate findings based on the data [27].

### Participants

Between April and June 2023, adult lesbian forced migrants were recruited through: (i) online advertisements (convenience sampling), (ii) the networks of the research team (purposeful sampling), and (iii) asking participants to inform others about the study (snowball sampling). Broad inclusion was applied to strive towards diversity in the sample. Thus, the following inclusion criteria were applied: (i) self-identifying as lesbian or bisexual, (ii) experience of being a forced migrant, regardless of current legal status (e.g., undocumented migrant, asylum seeker, refugee, permanent residence), and (iii) adult 18 years or older. Participants expressed an interest via a digital application form or by calling a research assistant. A time and place for an interview was determined depending on their preferences. The final sample consisted of 10 persons self-identifying as lesbian and one self-identifying as bisexual. The participants were between the ages of 24 and 45 years (median: 37). Highest level of education included university/college (*n* = 8), high school (*n* = 2), and elementary school (*n* = 1). Ten originated from Uganda and one originated from Sudan. Time in Sweden since arrival was less than one year (*n* = 7), one year (*n* = 3), and two years (*n* = 1). Nine identified as cisgender women and two did not want to define their gender.

### Data collection

The first and last authors conducted semi-structured interviews with an interview guide (Table 1). Based on participant preferences, nine interviews were conducted in English and two were conducted in Luganda utilizing a research assistant as the interpreter. The research assistant was familiar with terminology in sexual and gender minorities and spoke fluently in both languages. According to the preferences of the participants, five interviews were carried out at a hotel, five at the university campus where the study was conducted, and one via a digital video conferencing tool. Interview length ranged between 32 and 120 min (median: 68). All were audio-recorded and transcribed verbatim. The interviews conducted in Luganda were transcribed by a research assistant and confirmed by another research assistant, both fluent in Luganda and English. The interviewers are researchers and registered nurse-midwives, self-identifying as a woman and genderqueer, respectively. An expert by lived experience attended a proportion of interviews as an observer, to facilitate communication, promote participant comfortability, take notes, and ask follow-up questions. The observer was part of the analysis team presented below.

**Table 1:** Interview guide

Topic	Main question	Follow-up questions
Psychosocial health and wellbeing	How has your situation been since you came to Sweden?	How has your psychological health been?
		How has your physical health been?
		What has been difficult for you since you arrived?
		What larger challenges have you encountered?
		How would you describe your social situation from when you first arrived in Sweden up until today?
Psychosocial support	What kind of support have you experienced during your time in Sweden?	What have you done to make yourself feel good or to improve your health and wellbeing?
		Have you received the support you needed?
		What kind of support do you feel has been missing?
		Do you have any examples when you didn’t receive the support you needed from health services?
		Have you ever felt discriminated against when in contact with health services, and if so, in what ways?
Competence in health services	How have you experienced the contact with and the treatment by health services/health professionals?	What do you think is important for health professionals to consider when they support sexual minority forced migrants?
		Do you think health professionals have enough knowledge about sexual minorities and forced migrants?
Areas for improvement and interventions	How do you think the support for sexual minority forced migrants can be improved in Sweden?	What could be improved in the contact and treatment with health services/health professionals?
		What kind of support would have been good for you to receive after arriving in Sweden?
		How do you feel health services can be improved to better meet the support needs of sexual minority forced migrants?

### Data analysis

A collaborative approach was applied during analysis, involving a team of two researchers, two experts by lived experience, and one clinical psychologist. The material was analyzed iteratively according to the four steps in systematic text condensation [29]. In the first step, preliminary themes were identified. The analysts read all transcripts and identified preliminary themes, before collaborating to produce joint versions. In the second step, meaning units, defined as fragments of text containing information corresponding to the aim, were identified and placed into code groups. Following individual identification and coding of meaning units, analysts collaborated to scrutinize the units and revised the coding through an iterative process, In the third step, meaning units were sorted into subgroups, which were identified individually before formalized jointly. Analysts then proceeded to write individual condensates, defined as artificial quotes derived from the meaning units within a given subgroup, which were reviewed in joint meetings to produce final versions. Reviewing the raw data, analysts collaborated to find illustrative quotes portraying the content of each subgroup. In the fourth step, analysts collaborated to write synthesized statements based on the condensates and produced category headings serving as brief and expressive statements of the most significant findings. Please see an example of the analytic procedure in Table 2. Supplemental File 1 presents an expanded description of the analytic process. Systematic text condensation is an approach for cross-case thematic analysis leading to synthesized findings based on qualitative data such as interviews. Inspired by phenomenological analyses, systematic text condensation presents a pragmatic method through a stepwise process [29]. Based on our experiences, it is a suitable strategy when co-analyzing data together with experts by lived experience.

**Table 2:** Example of the analytic process, from meaning units to category heading

Meaning unit	Subgroups	Code group	Category heading
“It wasn’t easy. It’s not easy still, because I’ve been moving from house to house.”	The fragility of new beginnings as forced migrant women struggle to secure basic needs	Living in uncertainty	Carrying shackles of the past while fearing the future: living in unsafety and uncertainty
“Very difficult because here in Sweden I’m living here on favors. I never imagined that the day will come, and I sit for one year without doing anything.”			
“The interpreter I was given had never interpreted for LGBTQ case, so, my lawyer said ‘no, I won’t use him, and they could easily spoil your case’, so I have had two attempts of interviews.”	Opening emotional wounds and fearing deportation during asylum process		
“Sometimes it really stresses because being undercover is not really comfortable, you know.”			

Reflexivity was approached through various strategies. The team brought diverse positionalities and backgrounds that influenced the analysis. The two researchers who interviewed the participants were involved throughout all steps of the analysis, contributing with perspectives as health professionals and non-migrant researchers. The principal investigator self-identifies as a member of the sexual and gender minority community. Two experts by lived experience collaborated as team members throughout all steps of the analysis, contributing with perspectives as persons with lived experience as sexual and gender minority forced migrants. These experts by lived experience were instrumental in grounding the findings in insider perspectives, validating the procedure by bringing in in-depth insights based on their unique perspectives. One psychologist contributed with clinical perspectives into mental health, trauma, and coping. The team collaborated throughout the steps until all agreed that the findings reflected the interviews. The diverse expertise within the team shaped the findings. For example, the Swedish-born researchers highlighted aspects related to health services and structural inequities in Swedish society, the experts by lived experience highlighted aspects related to lived experiences of discrimination and marginalization, and the clinical psychologist highlighted aspects related to psychological distress and symptomatology. Reflexive meetings were held continuously to challenge preconceptions and delve into the data collaboratively. The researchers continuously reflected on their privileged perspectives, striving to protect the perspectives of the experts by lived experience to as high extent as possible. The researchers positioned themselves as having an outside view on the data and were careful to impose on the integrity of the findings by influencing it based on their assumptions and theoretical perspectives. The experts by lived experience were closely involved as equal partners in the research team, engaged in all steps of the analysis.

## Results

**Fig. 1:**
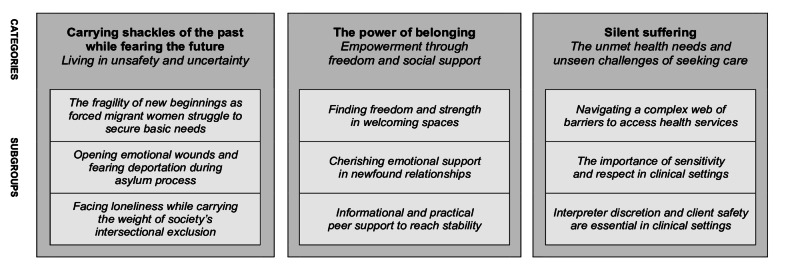
Overview of the main findings illustrating the psychosocial health among lesbian forced migrants

The analysis resulted in three main categories (Fig. 1). Following their arrival in Sweden, lesbian forced migrants experienced a challenging journey involving intersectional disadvantages and unsafe living situations. A newfound freedom and social support empowered them, as they found strength in welcoming spaces and belonging among peers. When trying to access health services, participants encountered barriers and non-affirming behaviors. Interpreters’ discretion and safety were considered essential aspects when they are utilized in health services.

### Carrying shackles of the past while fearing the future: living in unsafety and uncertainty

The first category concerns how lesbian forced migrants of African origin experience an unsafe and uncertain living conditions in the host country, illuminating the intersection of pre-migration trauma and post-migration stressors. It portrays the significant challenges encountered in the host country, whilst experiencing exclusion and loneliness. Key areas related to minority stress are reflected in this category, including the distal and proximal minority stress processes that impact the health and wellbeing of sexual and gender minority individuals. The category is illustrated through three subgroups highlighting their intersectional challenges and disadvantages whilst trying to acclimatize to a new and unfamiliar society: (i) the fragility of new beginnings as forced migrant women struggle to secure basic needs (ii) opening emotional wounds and fearing deportation during asylum process, and (iii) facing loneliness while carrying the weight of society’s intersectional exclusion.

#### The fragility of new beginnings as forced migrant women struggle to secure basic needs

Upon arrival in Sweden, participants found themselves in stressful and unsafe circumstances, underscoring the vulnerable situation exacerbated by marginalization and migration-related stressors. Due to international regulations, some had to live as undocumented for an extended period, exposing them to dangerous conditions with a risk of sexual exploitation. The housing situation had been unstable for several, involving temporary solutions and a risk of eviction with short notice. While asylum accommodations provided more stability, sharing small rooms with many other migrants had been stressful. Being exposed as a sexual and gender minority individual further compounded the already vulnerable situation, with participants’ stories illustrating how they had been rejected by people and left to fend for themselves alone.

*When I came to stay with this family*,* they didn’t know I am lesbian. So*,* when they got to know about my sexual orientation*,* they told me they can’t live with me. So now here I am*,* I’m stranded and I’m looking for accommodation.* (Participant A)

Participants struggled with significant financial challenges, lacking the means for basic sustenance. Restrictions in the labor market, coupled with discrimination and language barriers, resulted in difficulties finding a reliable employment. This forced several into vulnerable working conditions through under-the-table employment or earning money informally through baby-sitting.

*I cannot support myself*,* yet they expect me to get the job and yet to get that job*,* I need to first learn the Swedish language. So*,* I cannot really support myself because of the situation of the language*,* yeah and getting a job. [Interviewer: So*,* getting a job is another challenge then.] Because I was even told to get a job after my first interview*,* but it’s not easy to get a job right since December when I did my first interview*,* it’s not easy to get a job here unless you know Swedish*,* that why now am struggling to learn Swedish. (Participant B)*

#### Opening emotional wounds and fearing deportation during asylum process

Going through the asylum process had been a considerable emotional undertaking, involving repeated and lengthy asylum interviews that exacted a heavy toll on mental health. Prolonged periods of uncertainty, coupled with the fear of being forced to relocate if denied asylum, exacerbated anxiety and unrest. The findings reflect how the asylum system can perpetuate structural oppression and minority stress. The asylum process was a significant source of further minority stress, as participants were expected to openly talk about intimate information they had kept hidden throughout their lives. This reliving of painful memories opened emotional wounds and was experienced as a profoundly stressful event.

*Who you really are is something you’ve pushed down back there and learned new ways to hide it. Now [during asylum interviews] you have to bring it back to life […] it’s a bit nerve-wrecking*,* you don’t know if they will accept you and then it’s your life on the line.* (Participant C)

The importance of adequate interpretation services during asylum interviews was underscored, as participants feared that mistranslations could heavily influence decisions and lead to dire consequences. Moreover, participants needed to wait long periods in uncertainty before receiving a decision, which exacerbated mental health burdens. Some had faced additional challenging periods in uncertainty when living as undocumented. Facing a potential denial and consequently having to return to the dangerous circumstances in their country of origin was a terrifying thought.

*What runs through the head [while living undocumented]*,* ‘what is going to happen’*,* watching your back all the time. You sit on the train*,* and you think*,* “okay what’s going to happen”. So much like… I don’t know how I can describe it. You can still live your life even in the six months but the thought of if they get to know I am here*,* because anything can happen. […] It’s [like] being in the prison. It is. Because I mean there is nothing for you and if you are in this prison and you don’t have information about where to go for help it’s even worse.* (Participant D)

#### Facing loneliness while carrying the weight of society’s intersectional exclusion

While some participants had not encountered discrimination in Sweden, others recounted experiences of intersectional discrimination and violence based on both homophobia and racism/xenophobia. The experiences illustrate the risk of encountering proximal minority stress processes when having a migrant background. Having multiple converging minority statuses involved a risk of further exploitation and abuse. Participants feared being exposed to violence based on homophobia enacted by other forced migrants, landlords, and flat mates.

*You have now another stress of being in homophobic area where you are being mistreated. We need love*,* we need a warm hug. Yes*,* we are adults but then we need people to remind us that we are loved*,* that we matter. That we are normal humans. […] Then you go to a homophobic camp.* (Participant D)

Loneliness and social exclusion had a significant negative impact on health and wellbeing. Language barriers, the need to conceal their sexual orientation, and the fear to socially engage with others contributed to difficulties forming meaningful and supportive relationships. Several desperately missed their partners still trapped in their country of origin, further compounding psychological distress and loneliness. Several of the women had children whom they hoped could join them in Sweden once their life circumstances improved. Being separated from their children, they feared for their children’s safety in their country of origin.

*When I arrived here in Sweden everything was very difficult for me because I had no one here. I had no family*,* no friend*,* I had no one to trust so it was very difficult.* (Participant E)

### The power of belonging: empowerment through freedom and social support

The second category concerns the positive impact of finding freedom and social support, reflecting the empowerment and wellbeing cultivated through newfound freedom and community engagement leading to social support. The narratives shared by the participants illustrate the potential power of social support as a source that can ameliorate minority stress. The category is further elucidated in three subgroups: (i) finding freedom and strength in welcoming spaces, (ii) cherishing emotional support in newfound relationships, and (iii) informational and practical peer support to reach stability.

#### Finding freedom and strength in welcoming spaces

Participants expressed a deep appreciation of the freedom and rights provided for lesbians in Sweden, which was in stark contrast to the oppression they had faced in their country of origin. Witnessing lesbian couples being openly affectionate and participating in Pride was a joyful and eye-opening experience, mitigating the sense of exclusion and minority stress. Feeling free to express themselves and to live more authentic lives, participants expressed that they belonged in Sweden and felt at home.

*I got here [in Sweden] and I was accepted*,* I was welcomed. I saw people like me in the streets*,* in the trains. Yeah*,* it was very amazing. And I thought yes*,* this was going to work out for me.* (Participant F)

Participants felt hopeful about their future, desired to establish themselves in the society, wanted to gain independence, and hoped that their partner could join them in Sweden. For strength and to cope with post-migration stress, participants turned to their faith, engaged in hobbies, were physically active, and engaged in other recreational activities. They acknowledged the progress they had made and felt able to leave homophobic people behind, now taking steps towards safety and to rebuild their lives.

*I taught myself how to paint. […] You can express everything that you cannot tell anyone on that canvas*,* and then you will be free. Anger*,* depression*,* happiness*,* love*,* everything. Just put it on canvas. So*,* that’s when I taught myself how to use canvas and paint*. (Participant C)

#### Cherishing emotional support in newfound relationships

To counteract the social isolation and loneliness inherently associated with minority stress, participants actively sought company and community through social networks, non-governmental organizations, and churches. Several found meaningful relationships with likeminded friends who embraced them and accepted them as they are. Emotional support from friends made participants feel validated and welcomed in social settings where they felt they belonged. Peer support involved sharing of stories, cultural exchange, and guidance. Participants expressed that being in the company of peers felt like being released from jail and that they found themselves in an inclusive safe space.

*Psychologically it has been a lot of help. You get to meet other people and know you’re not alone. You talk about your experiences with them*,* you share… they also share their own experiences. You get to listen to other people’s stories. How they came to come here*,* what they went through before they came here. Then you also look at your own experience and you’re like*,* I think I’m not alone. And somehow*,* we manage to talk to each other and console each other. It has been of much help*,* I should say.* (Participant A)

Peer support brought emotional comfort and reduced the sense of isolation when facing significant challenges. Bonding with peers was likened to finding a second chosen family. Participants also expressed a desire to help peers and give back to the community by volunteering at non-governmental organizations.

*Everyone wants to help because when we are there in our community*,* we are like a family. You feel safe and you can say anything*,* because you know you are in your right position*,* where you belong. So*,* it’s like our home.* (Participant E)

#### Informational and practical peer support to reach stability

Participants attended different activities arranged by non-governmental organizations, which involved the exchange of informational and instrumental support contributing to stability in their daily lives. Gaining information about a range of topics meant an opportunity to understand unfamiliar and complex structures in society. Information on the migration process and sexual health was highly appreciated.

*Because as we always meet*,* in a week*,* they [at non-governmental organization] teach us on how to socialize*,* on how to fight stress*,* and we meet other people*,* and we discuss different kinds of ideas they give me and how to handle… they generally tell us how life should be*,* how life is handled for [sexual and gender minority] people in Sweden.* (Participant B)

Highly valued instrumental support was also attained through peer support, including food, transportation, clothing, interpretation, and physical activities. Several appreciated the support provided by legal advisors they gained contact with through the non-governmental organizations, who helped them prepare for asylum interviews and offered information about the legal processes. The legal support boosted the confidence of participants and empowered them to talk about the difficult things brought up during asylum interviews.

*[Non-governmental organization] provide us with legal advisors. And the legal advisors*,* they tell you your rights as an asylum seeker […] She helps to prepare you for the interview. […] Trust me*,* she pushed it. Like*,* I talked to her three times in a week*,* physical. And she’d always tell me ‘You’re improving. The first time you didn’t tell me anything*,* you didn’t do nothing’*,* you know. But with time*,* I learned. I have. Still*,* it breaks me down*,* but at least I could talk.* (Participant G)

### Silent suffering: the unmet health needs and unseen challenges of seeking care

The third category illuminates the substantial unmet health needs and unseen challenges of seeking care in the host country, highlighting how structural barriers and fears impede and limit healthcare access. It illustrates how access was hindered by both external and internal processes, involving an intricate dynamic of dimensions related to shame, identity, migration status, and disclosure. The category is constructed by three subgroups: (i) navigating a complex web of barriers to access health services, (ii) the importance of sensitivity and respect in clinical settings, and (iii) interpreter discretion and client safety are essential in clinical settings.

#### Navigating a complex web of barriers to access health services

Participants described a range of structural barriers that hindered their access to needed health services, exemplifying the systemic inequities encountered by forced migrants. Financial constraints led to some not receiving care or medication despite needing it, particularly when living as undocumented with limited resources. Access to free health services was highly appreciated. Language barriers, bureaucratic hurdles, documentation requirements, and a lack of familiarity with the healthcare structure in Sweden further contributed to the difficulty to schedule appointments with health professionals.

*The health system is difficult from one thing that you have to call and book an appointment. But the telephone is speaking in Swedish. […] You have to insist. Because they tell you “you didn’t book an appointment”. You have to beg and say please.* (Participant E)

Once they were able to book an appointment, participants experienced frustratingly long waiting before receiving care. Participants feared that they would encounter discrimination and be reported to authorities if seeking care. This led to hesitation about whether health services could be trusted. A fear of undergoing gynecological screening in clinical settings was expressed. Home-based HPV-testing was seen as a positive alternative, although some uncertainty remained about the reliability of the results due to the self-sampling method. Participants who were unable to access healthcare through formal routes felt they needed to beg for help from health professionals and turned to the Internet for guidance.

[Interviewer: Did you get any information from somewhere about the healthcare system. Where to turn.] From a friend. She told me like, if you want to have access to healthcare here, you have to go through a system. You don’t just go to a pharmacy, you don’t just go to a hospital. […] It’s not easy to access medication, healthcare. But sometimes if it’s really, really hard and you’re really going through maybe a lot of pain and [can’t] communicate. (Participant H)

#### The importance of sensitivity and respect in clinical settings

Sensitivity and respect in clinical settings were identified as a key area for health services, highlighting the importance of affirming encounters with health professionals. Participants appreciated when they were supported by health professionals who were welcoming, respectful, and comforting. Whilst some had only positive experiences of interactions with health professionals, others recounted experiences of dismissive and disrespectful behaviors. Participants had experienced these instances as associated with both their status as migrants and homophobia. These instances further contributed to minority stress reactions.

*[Health professionals] saw me with the rainbow tag on my hand. So*,* I saw some signals from the receptionists. […] The expression and the communication*,* though it wasn’t verbal*,* but I could really tell that there is something that is not right. Either they are wondering or [knew they were] working on a lesbian*,* one [sexual and gender minority person]. Meaning they are still lacking some information. And not everyone is really informed.* (Participant H)

Some experienced considerable difficulties in obtaining the healthcare that they needed and were entitled to as undocumented migrants. Additionally, based on their previous experiences of health services and a fear of homophobic reactions, participants were apprehensive and hesitant to disclose information about their sexuality during interactions with health professionals in Sweden. They desired validating encounters in which questions about identity were asked in a non-judgmental and safe manner.

*I sit down with [medical doctor] and we talk just like I’m talking with you. I tell her things*,* like this is paining me. This*,* that*,* [inaudible] like she’s listening to me. And I’m just talking. I feel comfortable. At least I feel comfortable. [Interviewer: What do they do to make you feel comfortable.] She’s welcoming. She picks me from the reception*,* she waits for me*,* holds my hand. It always… that calms me down and I’m ready to open up and talk more*,* even when I’m not supposed to. I just feel comfortable. I like the comfort that they make me feel when I’m in her office.* (Participant F)

#### Interpreter discretion and client safety are essential in clinical settings

Several participants emphasized the importance of utilizing competent professional interpreters in clinical settings, to ensure high-quality and accurate communication. Safety and confidentiality were considered essential aspects when utilizing interpreters in health services. Some participants felt uncomfortable sharing intimate information when interpreters are present, highlighting the importance of sensitivity and affirming behaviors. Others mentioned positive experiences of interpreters, further highlighting the importance of confidentiality.

*I haven’t gotten any problem because even if that person is from [country of origin]*,* these people who bring this interpreter*,* they assure me that whatever we say ends here and by the time they bring that person*,* it means that they trust that person. So*,* I don’t expect information to go to [country of origin] because they assure me that it ends here*. (Participant I)

The utilization of interpreters originating from the same region or culture as the participants themselves raised concerns of encountering judgmental attitudes. Furthermore, some worried about the potential spread of rumors in the community when interpreters are used. Telephonic interpretation was considered a more anonymous alternative than face-to-face conversations, as it promoted a sense of safety.

*On the phone*,* it was good because I was hearing [interpreter] well. Secondly*,* that interpreter he cannot recognize me. Even if he would see me in another day*,* he will not know or see that this is the woman I was talking with. So*,* it’s like a secret. And so*,* I think it’s better if someone is on the phone. The interpreter*,* you can be free to tell them anything. You cannot be shy. There are some things maybe you cannot talk when someone is there and think ‘oh*,* how will this one say’. Remember*,* you come from the same country*,* and you know*,* the culture there*,* and then you are like ‘let me not share this’.* (Participant E)

## Discussion

This study makes conceptual contributions by offering in-depth insights into the psychosocial health of lesbian forced migrants, a population that has largely been unrecognized in research [17] and subsumed in research within the broader population of sexual and gender minority migrants [30, 31]. An intersectional perspective [32] taking into consideration the societal marginalization and discrimination against women, gender diverse people, migrants, and sexual minority individuals can bring further insights about the disadvantages faced by lesbian forced migrants. Our study illustrates exposure to violence stemming from both homophobia and racism or xenophobia, vividly illustrating the complex interplay between different marginalized identities. While this study provides initial insights, further research is needed to explore experiences through a clearer intersectional lens.

Our study underscores the risk of exploitation related to intersectional vulnerabilities rooted in sexual orientation, migrant status, and gender, echoing patterns observed in other sexual minority forced migrants [33, 34] and transgender women [35]. The unstable living conditions and lack of financial resources illuminated in this study further emphasize structural marginalization and severe challenges related to homelessness [36–38] and employment [20, 39]. The precarious living conditions and significant psychological distress shown in our findings calls attention to the mental [40] and physical [6] health problems associated with minority stress. The substantial burden of discrimination and victimization, as encountered by our participants, gives further weight to acknowledging sexual and ethnic minority individuals as a high-risk group for experiencing minority stress [40]. Taken together, there is a need to improve the support of lesbian forced migrants on a policy and structural level. Health professionals need to adopt an intersectional analytical lens to proactively screen for violence and mental health burdens, ensuring extended support and protection for those facing compounded marginalization and oppression.

Our study demonstrates how loneliness, a known public health concern associated with a range of severe negative health effects [41], shapes the lived experience and burdens among lesbian forced migrants. Forced migration damage social relationships [42], which may be further exacerbated for lesbian forced migrants by their intersecting identities [17, 43] and felt need to conceal their identity due to stigma-related stress [7]. While research collectively highlights an increased risk of loneliness among forced migrants and sexual minority individuals, few studies have reported about the experiences of sexual minority forced migrants [44]. Critically, our findings illuminate peer support as a counter-narrative to the profound loneliness, having the potential to offer a range of emotional benefits for underserved populations [45] such as sexual minority individuals [46] and forced migrant women [47]. While some studies have proposed social support as a valuable resource for emotional relief and affirmation [17], our study confirms the potential benefits for lesbian forced migrants. Peer support should be considered a potential intervention to mitigate stigma-related stress and promote resilience. However, more research is warranted to investigate the benefits of peer support interventions aiming to promote mental health within this population.

The evidence on health burdens experienced among forced migrants [16, 48] call attention to the importance of accessible healthcare during resettlement. While forced migrants are entitled to health services in many host countries such as Sweden, our findings show how structural barriers, lack of awareness, and discrimination [49] can render support inaccessible for lesbian forced migrants. These experiences seem to extend beyond the experiences of the general migrant population, as fears of encountering dismissive and disrespectful reactions based on one’s sexual orientation as lesbian. The findings expose a need to further improve adherence to ethical obligations in providing respectful and non-judgmental healthcare grounded in equity [50, 51]. While there is an overall lack of studies exploring how sexual minority forced migrants experience interactions with health professionals, our and two recent studies [52, 53] call attention to the occurrence of non-affirming practices within health services. Establishing a safe and inclusive clinical setting is essential when supporting sexual minorities [54, 55] and traumatized individuals [56]. Taken together, this study calls attention to the importance of upholding confidentiality and applying a person-centered approach when interpreters are utilized. Health professionals must be trained to recognize the compounded effects of minority stress and forced migration, integrating culturally sensitive and affirming approaches as needed. We encourage more research investigating cultural sensitivity when supporting lesbian forced migrants in health services.

There are methodological limitations of this study. Because this population is hard-to-reach through conventional routes, we utilized a combination of purposeful, convenience, and snowball sampling to reach a diverse sample. While we did not aim to only recruit participants from Uganda, the majority of participants originated from Uganda, one of the most oppressive countries for sexual minority individuals [2, 57]. We acknowledge that the findings to a higher extent reflect the experiences of persons from that country. Thus, the transferability to migrants from other countries may therefore be limited. To broaden the transferability, we also included one participant who self-identified as a bisexual woman. We encourage more research about the health and wellbeing of bisexual forced migrant women.

All interviews provided rich information that added to the findings. Based on participant preferences, one interview was conducted digitally. This interview was somewhat shorter but nevertheless provided valuable information. Two interviews were conducted in Luganda with the aid of a research assistant in the project, fluent in English and Luganda. The research assistant was familiar with terminology related to sexual and gender minorities. Another research assistant fluent in Luganda read the transcripts in Luganda and English and confirmed the translation. However, no back-translation was made to ensure fidelity, and thus, we cannot disregard the risk that information was lost due to the translation. We acknowledge the risk of semantic drift due to the translation, since certain nuances, words or phrases may not have the perfect one to one equivalent.

Readers should note that we did not conduct any member checking to enhance the credibility of the findings. This can be considered a limitation, since participants did not confirm our findings. We engaged in close collaboration with two experts by lived experience and one clinical psychologist who contributed to all analytic steps. While we argue that this nuanced and enriched the findings from additional perspectives [58], we cannot disregard the risk that some aspects may have been unintentionally left out. Throughout the analytic process, the team members engaged in mutual coding and held ongoing discussions to broaden their perspectives. The Swedish-born non-migrant researchers with prior experience of studies in this field collaborated with sexual and gender minority forced migrants to challenge their preconceptions. We believe that our collaborative approach strengthened the credibility of our findings. Nevertheless, we acknowledge that our joint positionalities and backgrounds shaped the findings. For example, the non-migrant researchers had an outside perspective that involved the risk of overlooking nuances that require other expertise. Conversely, the experts by lived experience provided lived experience perspectives that involved the risk of influencing the findings without basing assumptions on the participants’ descriptions. Based on the low number of qualitative studies focusing on lesbian forced migrants, readers should regard this study as an initial step towards context-based in-depth understandings about an underserved population.

## Conclusions

Lesbian forced migrants experience unsafe beginnings and persistent mental health burdens following their arrival in a host country. These challenges seem to be rooted in intersectional disadvantages and minority stress experienced in the post-migration phase. Loneliness is a prominent public health concern that may contribute to minority stress and additional mental health burdens. Access to welcoming spaces and communities where these women can engage in peer support has the potential to offer emotional and practical support that can mitigate loneliness and promote health. We encourage further research to explore the potential positive effects of social support on mental health.

Our findings underscore the risk that lesbian forced migrants are excluded from healthcare they need and are entitled to, contributing to further health burdens. Thus, there is a need for the implementation of policies that will improve their access to quality health services and achieve health equity. An important step is to improve health professionals’ awareness of minority stress and the psychosocial situation experienced by lesbian forced migrants. Ensuring sensitivity, respect, and discretion is essential when supporting these women in clinical settings. Refinement of policies, clinical guidelines, and clinical training seems needed, to further reduce the risk of non-affirming encounters in health services.

## Supplementary Information

Supplementary Material 1.

## Data Availability

The data are not publicly available because of reasons related to confidentiality.

## References

[1] Alessi EJ, Cheung S, Kahn S, Yu M. A scoping review of the experiences of violence and abuse among sexual and gender minority migrants across the migration trajectory. Trauma Violence Abuse. 2021;22(5):1339–55. 34812109 10.1177/15248380211043892

[2] Mendos LR, Botha K, Lelis RC, Peña ELdl, Savelev I, Tan D. State-sponsored Homophobia 2020: Global Legislation Overview Update. ILGA World; 2020.

[3] Yarwood V, Checchi F, Lau K, Zimmerman C. LGBTQI + migrants: a systematic review and conceptual framework of health, safety and wellbeing during migration. Int J Environ Res Public Health. 2022;19(2):869. 10.3390/ijerph19020869. 10.3390/ijerph19020869 PMC8775429 35055698

[4] Meyer IH. Prejudice, social stress, and mental health in lesbian, gay, and bisexual populations: conceptual issues and research evidence. Psychol Bull. 2003;129(5):674–97. 12956539 10.1037/0033-2909.129.5.674 PMC2072932

[5] Baptiste-Roberts K, Oranuba E, Werts N, Edwards LV. Addressing health care disparities among sexual minorities. Obstet Gynecol Clin North Am. 2017;44(1):71–80. 28160894 10.1016/j.ogc.2016.11.003 PMC5444328

[6] Flentje A, Heck NC, Brennan JM, Meyer IH. The relationship between minority stress and biological outcomes: a systematic review. J Behav Med. 2020;43(5):673–94. 31863268 10.1007/s10865-019-00120-6 PMC7430236

[7] Pitoňák M. Mental health in non-heterosexuals: minority stress theory and related explanation frameworks review. Ment Health Prev. 2017;5:63–73.

[8] Patanè M, Ghane S, Karyotaki E, Cuijpers P, Schoonmade L, Tarsitani L, Sijbrandij M. Prevalence of mental disorders in refugees and asylum seekers: a systematic review and meta-analysis. Glob Ment Health (Camb). 2022;9:250–63. 36618716 10.1017/gmh.2022.29 PMC9806970

[9] Blackmore R, Boyle JA, Fazel M, Ranasinha S, Gray KM, Fitzgerald G, et al. The prevalence of mental illness in refugees and asylum seekers: a systematic review and meta-analysis. PLoS Med. 2020;17(9):e1003337. 32956381 10.1371/journal.pmed.1003337 PMC7505461

[10] Hossin MZ. International migration and health: it is time to go beyond conventional theoretical frameworks. BMJ Glob Health. 2020;5(2):e001938. 32180999 10.1136/bmjgh-2019-001938 PMC7053782

[11] Jolof L, Rocca P, Mazaheri M, Okenwa Emegwa L, Carlsson T. Experiences of armed conflicts and forced migration among women from countries in the middle East, Balkans, and Africa: a systematic review of qualitative studies. Confl Health. 2022;16(1):46. 36071504 10.1186/s13031-022-00481-x PMC9450290

[12] Rubenstein BL, Lu LZN, MacFarlane M, Stark L. Predictors of interpersonal violence in the household in humanitarian settings: a systematic review. Trauma Violence Abuse. 2020;21(1):31–44. 29334000 10.1177/1524838017738724

[13] Heslehurst N, Brown H, Pemu A, Coleman H, Rankin J. Perinatal health outcomes and care among asylum seekers and refugees: a systematic review of systematic reviews. BMC Med. 2018;16(1):89. 29890984 10.1186/s12916-018-1064-0 PMC5996508

[14] Gottvall M, Sjölund S, Arwidson C, Saboonchi F. Health-related quality of life among Syrian refugees resettled in Sweden. Qual Life Res. 2020;29(2):505–14. 31617059 10.1007/s11136-019-02323-5 PMC6994443

[15] Hawkins MM, Schmitt ME, Adebayo CT, Weitzel J, Olukotun O, Christensen AM, Ruiz AM, Gilman K, Quigley K, Dressel A, et al. Promoting the health of refugee women: a scoping literature review incorporating the social ecological model. Int J Equity Health. 2021;20(1):45. 33485342 10.1186/s12939-021-01387-5 PMC7825239

[16] Lebano A, Hamed S, Bradby H, Gil-Salmerón A, Durá-Ferrandis E, Garcés-Ferrer J, Azzedine F, Riza E, Karnaki P, Zota D, et al. Migrants’ and refugees’ health status and healthcare in europe: a scoping literature review. BMC Public Health. 2020;20(1):1039. 32605605 10.1186/s12889-020-08749-8 PMC7329528

[17] Gottvall M, Brunell C, Eldebo A, Johansson Metso F, Jirwe M, Carlsson T. Post-migration psychosocial experiences and challenges amongst LGBTQ + forced migrants: a meta-synthesis of qualitative reports. J Adv Nurs. 2023;79(1):358–71. 36320151 10.1111/jan.15480 PMC10092230

[18] Danisi C, Dustin M, Ferreira M, Held N. Queering Asylum in Europe: Legal and Social Experiences of Seeking International Protection on grounds of Sexual Orientation and Gender Identity. Switzerland: Springer Nature; 2021.

[19] Schock K, Rosner R, Knaevelsrud C. Impact of asylum interviews on the mental health of traumatized asylum seekers. Eur J Psychotraumatol. 2015;6:26286. 26333540 10.3402/ejpt.v6.26286 PMC4558273

[20] Waite S, Ecker J, Ross LE. A systematic review and thematic synthesis of Canada’s LGBTQ2S + employment, labour market and earnings literature. PLoS One. 2019;14(10):e0223372. 31577824 10.1371/journal.pone.0223372 PMC6774499

[21] Crenshaw K. Demarginalizing the intersection of race and sex: a black feminist critique of antidiscrimination doctrine, feminist Eory and antiracist politics. Univ Chic Legal F. 1989;1(8):139–67.

[22] Tschalaer M. Queering migration temporalities: LGBTQI + experiences with waiting within Germany’s asylum system. Ethnic Racial Stud. 2023;46(9):1833–53.

[23] Bennett C, Thomas F. Seeking asylum in the UK: lesbian perspectives. Forced Migr Rev. 2013;42:25–8.

[24] Chaudhry A, Hebert-Beirne J, Alessi EJ, Khuzam MZ, Mitchell U, Molina Y, et al. Exploring the health impact of intersectional minority identity stressors on Arab sexual minority women migrants to the United States. Qual Health Res. 2025;35(3):305–18. 39172019 10.1177/10497323241265288

[25] Hord LC. Specificity without identity: articulating post-gender sexuality through the non-binary lesbian. Sexualities. 2022;25(5–6):615–37.

[26] Salsberg J, Parry D, Pluye P, Macridis S, Herbert CP, Macaulay AC. Successful strategies to engage research partners for translating evidence into action in community health: a critical review. J Environ Public Health. 2015;2015:191856. 10.1155/2015/191856. 10.1155/2015/191856 PMC4359847 25815016

[27] Patton MQ. Qualitative Research & Evaluation Methods: Integrating Theory and Practice. 4th ed. California: SAGE Publications; 2015.

[28] Vargas SM, Huey SJ, Miranda J. A critical review of current evidence on multiple types of discrimination and mental health. Am J Orthopsychiatry. 2020;90(3):374–90. 31999138 10.1037/ort0000441

[29] Malterud K. Systematic text condensation: a strategy for qualitative analysis. Scand J Public Health. 2012;40(8):795–805. 23221918 10.1177/1403494812465030

[30] Lee EOJ, Brotman S. Identity, refugeeness, belonging: experiences of sexual minority refugees in Canada. Can Rev Sociol. 2011;48(3):241–74. 22214042 10.1111/j.1755-618x.2011.01265.x

[31] Logie CH, Lacombe-Duncan A, Lee-Foon N, Ryan S, Ramsay H. It’s for us -newcomers, LGBTQ persons, and HIV-positive persons. You feel free to be: a qualitative study exploring social support group participation among African and Caribbean lesbian, gay, bisexual and transgender newcomers and refugees in Toronto, Canada. BMC Int Health Hum Rights. 2016;16(1):18. 27369374 10.1186/s12914-016-0092-0 PMC4930565

[32] Hill Collins P, Bilge S. Intersectionality. Oxford, United Kingdom: polity; 2016.

[33] Wimark T. Homemaking and perpetual liminality among queer refugees. Soc Cult Geogr. 2021;22(5):647–65.

[34] Karimi A. Sexuality and integration: a case of gay Iranian refugees’ collective memories and integration practices in Canada. Ethnic Racial Stud. 2021;44(15):2857–75.

[35] Cerezo A, Morales A, Quintero D, Rothman S. Trans migrations: exploring life at the intersection of transgender identity and immigration. Psychol Sex Orientat Gend Divers. 2014;1(2):170–80.

[36] Fraser B, Pierse N, Chisholm E, Cook H. LGBTIQ + homelessness: a review of the literature. Int J Environ Res Public Health. 2019;16(15):2677. 10.3390/ijerph16152677. 10.3390/ijerph16152677 PMC6695950 31357432

[37] Samari D, Groot S. Potentially exploring homelessness among refugees: a systematic review and meta-analysis. J Soc Distress Homelessness. 2023;32(1):135–50.

[38] Phipps M, Dalton L, Maxwell H, Cleary M. Women and homelessness, a complex multidimensional issue: findings from a scoping review. J Soc Distress Homelessness. 2019;28(1):1–13.

[39] Kia H, Robinson M, MacKay J, Ross LE. Poverty in Lesbian, Gay, Bisexual, Transgender, Queer, Two-Spirit, and other sexual and gender minority (LGBTQ2S+) communities in Canada: implications for social work practice. Res Soc Work Pract. 2021;31(6):584–98. 34475728 10.1177/1049731521996814 PMC8404727

[40] Sattler FA, Zeyen J. Intersecting indentities, minority stress, and mental health problems in different sexual and ethnic groups. Stigma Health. 2021;6(4):457.

[41] Park C, Majeed A, Gill H, Tamura J, Ho RC, Mansur RB, Nasri F, Lee Y, Rosenblat JD, Wong E, et al. The effect of loneliness on distinct health outcomes: A comprehensive review and Meta-Analysis. Psychiatry Res. 2020;294:113514. 33130511 10.1016/j.psychres.2020.113514

[42] Ekoh PC, Iwuagwu AO, George EO, Walsh CA. Forced migration-induced diminished social networks and support, and its impact on the emotional wellbeing of older refugees in Western countries: A scoping review. Arch Gerontol Geriatr. 2023;105:104839. 36343437 10.1016/j.archger.2022.104839

[43] Piwowarczyk L, Fernandez P, Sharma A. Seeking asylum: challenges faced by the LGB community. J Immigr Minor Health. 2017;19(3):723–32. 26976005 10.1007/s10903-016-0363-9

[44] Warr D, Cox J, Redshaw S. A review of associations between social isolation, loneliness and poor mental health among five population groups. New South Wales: Murrumbidgee Primary Health Network; 2020.

[45] Sokol R, Fisher E. Peer support for the hardly reached: a systematic review. Am J Public Health. 2016;106(7):e1-8. 27196645 10.2105/AJPH.2016.303180 PMC4984766

[46] McDonald K. Social support and mental health in LGBTQ adolescents: a review of the literature. Issues Ment Health Nurs. 2018;39(1):16–29. 29333899 10.1080/01612840.2017.1398283

[47] Guruge S, Thomson MS, George U, Chaze F. Social support, social conflict, and immigrant women’s mental health in a Canadian context: a scoping review. J Psychiatr Ment Health Nurs. 2015;22(9):655–67. 26031541 10.1111/jpm.12216

[48] Kumar GS, Beeler JA, Seagle EE, Jentes ES. Long-term physical health outcomes of resettled refugee populations in the United States: a scoping review. J Immigr Minor Health. 2021;23(4):813–23. 33515162 10.1007/s10903-021-01146-2 PMC8233239

[49] Mangrio E, Sjögren Forss K. Refugees’ experiences of healthcare in the host country: a scoping review. BMC Health Serv Res. 2017;17(1):814. 29216876 10.1186/s12913-017-2731-0 PMC5721651

[50] The ICN code. of ethics for nurses [https://www.icn.ch/sites/default/files/inline-files/ICN_Code-of-Ethics_EN_Web.pdf]

[51] WMA International Code of Medical Ethics. [https://www.wma.net/policies-post/wma-international-code-of-medical-ethics/]

[52] Haghiri-Vijeh R. Experiences of LGBTQIA + migrants with nurses and other healthcare professionals in Canada. Nurs Forum. 2022;57(6):1184–92. 36285823 10.1111/nuf.12819

[53] Carlsson T, Isaac R, Ainembabazi R, Eldebo A, Yasin S, Gottvall M. Desiring support on a winding road with challenging intersections: Social and professional support for sexual minority forced migrant men. J Adv Nurs. 2025;81(2):897–908. 10.1111/jan.16256. 10.1111/jan.16256 PMC11730383 38808511

[54] Mukerjee R, Wesp L, Singer R, Menkin D. Clinician’s Guide to LGBTQIA + Care: Cultural Safety and Social Justice in Primary, Sexual, and Reproductive Healthcare. 1 edn. New York: Springer Publishing Company; 2021.

[55] Brooks H, Llewellyn CD, Nadarzynski T, Pelloso FC, Guilherme FDS, Pollard A, Jones CJ. Sexual orientation disclosure in health care: a systematic review. Br J Gen Pract. 2018;68(668):e187–96. 29378698 10.3399/bjgp18X694841 PMC5819984

[56] SAMHSA. SAMHSA’s Concept of Trauma and Guidance for a Trauma-Informed Approach. In. Rockville, MD; 2014.

[57] McQuaid K. There is violence across, in all arenas’: listening to stories of violence amongst sexual minority refugees in Uganda. Int J Hum Rights. 2020;24(4):313–34.

[58] Flicker S, Nixon SA. The DEPICT model for participatory qualitative health promotion research analysis piloted in Canada, Zambia and South Africa. Health Promot Int. 2015;30(3):616–24. 24418997 10.1093/heapro/dat093 PMC4542917

