# Supplementary material for: ‘We need a warm hug to remind us that we are loved’: a qualitative study of psychosocial health and wellbeing among lesbian forced migrants
Source: BMC Public Health. 2026 Jan 17;26:778. doi: 10.1186/s12889-026-26240-8 (PMC12958516; doi:10.1186/s12889-026-26240-8)
Supplement: Supplementary file 1 — Supplementary Material 1. [file 12889_2026_26240_MOESM1_ESM.pdf]

Additional File 1. Description of the analytic process according to systematic text condensation presented by Malterud [1].

Identification of preliminary themes

Description of analytic step

Full transcripts are read to gain an overall impression of the data. Data is approached with an open mind, trying to capture the voices of the participants. Having read the transcripts, preliminary themes are identified. Analysis benefits by having more than one analyst involved, to create a wide analytic space. Preliminary themes are considered starting points for sequential data organization, intended to build further elaboration on.

Analytic process

The analysts involved in this study all read transcripts and identified their own preliminary themes individually. Following the individual identification, joint meetings were arranged to discuss commonalities and differences between the preliminary themes of the analysts. All analysts collaborated to produce joint versions of preliminary themes.

Coding of meaning units into code groups

Description of analytic step

Transcripts are reviewed line by line to identify meaning units. Meaning units are defined as fragments of text containing information corresponding to the aim of the study. Sections of the transcript not answering the study aim are disregarded. Meaning units are then placed into code groups derived from the preliminary themes. The titles and content of code groups are iteratively contested and revised as the analysis progresses.

Analytic process

Analysts carefully scrutinized transcripts and marked meaning units, line-by-line, individually with marking pens. Their meaning units were cut out and placed into envelopes, each envelope corresponding to a code group. Joint meetings were held to scrutinize the meaning units and discuss the sorting into code groups from diverse perspectives. Code groups were revised iteratively according to the discussions.

Sorting meaning units into subgroups and writing condensates with illustrative quotes

Description of analytic step

Each code group is divided into subgroups illustrating specific content within the corresponding code group. Meaning units in each subgroup are reviewed and reduced into a condensate. Condensates are artificial quotations derived from the meaning units, maintaining the wording of the raw data as far as possible. Condensates are written in first-person format to represent cross-case information based on the complete collected data. Illustrative quotes from the raw data are identified to portray the content of each subgroup.

Analytic process

Analysts identified subgroups individually, followed by joint meetings to discuss and refine subgroups further. Once analysts all agreed on the identified subgroups, they collaboratively placed meaning units into the corresponding subgroups. Condensates were then written individually. All condensates were reviewed in joint meetings and analysts collaborated to write final versions encompassing all condensates within a given subgroup. Analysts reviewed the raw data individually to identify illustrative quotes, which were reviewed jointly until analysts agreed on one illustrative quote for each subgroup.

Writing synthesized statements and producing category headings

Description of analytic step

Data are reconceptualized as synthesized statements providing elucidation of the study aim. The synthesized statements are built based on the condensates generated in the previous analytic step. Statements are written in a narrative third-person format through the lens of interpretation. Examples drawn from the raw data are identified to further build on the synthetization. Previous analytic steps are reviewed iteratively by going back and forth to previous analytic steps. Category headings are finally produced to provide brief yet expressive statements of the most significant findings.

Analytic process

Analysts collaborated in joint meetings to produce synthesized statements derived from the condensates generated in the previous step. Synthesized statements were collaboratively scrutinized and revised by carefully returning to the meaning units and raw data. All analysts identified suggestions on category headings individually. All suggestions were then discussed in joint meetings until final versions were produced through the discussions.

References

1. Malterud K: **Systematic text condensation: a strategy for qualitative analysis**. *Scand J Public Health* 2012, **40**(8):795-805.
